# Why are Birth Defects Surveillance Programs Important?

**DOI:** 10.3389/fpubh.2021.753342

**Published:** 2021-11-02

**Authors:** Débora Gusmão Melo, Maria Teresa Vieira Sanseverino, Thanyse de Oliveira Schmalfuss, Mariela Larrandaburu

**Affiliations:** ^1^Department of Medicine, Federal University of São Carlos (UFSCar), São Carlos, Brazil; ^2^School of Medicine, Pontifical Catholic University of Rio Grande do Sul, Porto Alegre, Brazil; ^3^Medical Genetics Service, Clinical Hospital of Porto Alegre, Porto Alegre, Brazil; ^4^School of Medicine, Federal University of Rio Grande do Sul (UFRGS), Porto Alegre, Brazil; ^5^Disability and Rehabilitation Program, Ministry of Public Health of Uruguay, Montevideo, Uruguay

**Keywords:** birth defects, congenital anomalies, public health surveillance, epidemiological monitoring, registries, teratogens, newborn screening

## Introduction

Public health surveillance is described as the ongoing, systematic collection, analysis, and interpretation of health-related data essential to planning, implementation, and evaluation of public health policies strongly integrated with the opportune dissemination of these data to those responsible for prevention and control (1). Specifically, concerning birth defects or congenital anomalies, it is important to highlight the British Columbia Health Surveillance Registry, which has recorded cases of birth defects, genetic diseases, and chronic disabilities since 1952 (2), although the ascertainment sources and reporting procedures for birth defects have been more consistent since 1966 (3). Forsooth, public health surveillance of birth defects was driven by the thalidomide epidemic (4), which disclosed the need to establish systems that could identify teratogenic agents. More than 50 years later, the Zika epidemic reiterated this requirement (5, 6).

Congenital anomalies, defined as abnormalities of body structure or function that have a prenatal origin and are evident or not at birth, are a diverse group that can be caused by chromosomal disorders, single-gene defects, multifactorial inheritance, environmental teratogens or micronutrient malnutrition, and maternal illness (7). In 2004, an estimated 260,000 deaths globally were attributable to congenital anomalies. In 2010, the 63rd World Health Assembly adopted a resolution urging countries to develop and strengthen congenital anomalies surveillance systems (8).

Birth defects surveillance programs can usually be classified into two main types: population-based, which investigates birth defects among the whole population residing in a delimited geographic area, or hospital-based, which investigates birth defects in selected hospitals, maternity hospitals, or facilities, and which coverage corresponds to births or hospital admissions in these places (9). Concerning cases detection, it can be further divided into active case-finding, which requires systematic screening and clinical evaluation of children; passive case-finding, when affected individuals have access to health facilities and then are recognized; or a hybrid case-finding system (10). In addition to population coverage and case-finding, the design and data gathering on birth defects can be different among several surveillance programs mainly regarding the case definition, age of inclusion, inclusion or absence of data from prenatal diagnosis and elective termination of pregnancy for fetal anomaly (ETOPFA), congenital anomalies description, and coding systems (7). Although the definition of birth defects includes both structural and functional anomalies, birth defects surveillance programs often monitor major structural birth defects and sometimes minor structural birth defects, too (11). The detection of functional anomalies as inborn errors of metabolism and blood disorders is frequently performed by neonatal screening programs (8). In any case, the most significant aspect of a public health surveillance program is how the data collected will be used to promote the health of the people and the population (12).

In this opinion article, we discuss why birth defects surveillance programs are important and how they can track, assess, and improve the management of congenital anomalies at both the individual and collective levels, which makes a strong argument for continuing to monitor congenital anomalies around the world.

## Purposes and Objectives of Birth Defects Surveillance Programs

### Epidemiological Purpose

A regular purpose of birth defects surveillance programs is to provide epidemiologic data. This surveillance can point out baseline rates and monitor the trends in birth defects occurrence. Monitoring reveals quantitative estimates of the magnitude of the disease. In this way, surveillance programs can identify clusters of congenital defects and serve as an early detection system for unexpected increases in their frequency resulting from the introduction of new and old teratogens in the population (12–14). For example, this was how misoprostol and the Zika virus were related to a cluster of birth defects and identified as teratogens initially (15, 16). This is also how the cases of fetal rubella syndrome have been monitored and controlled in many regions around the world until today (17, 18).

### Planning and Prevention Purposes

Another purpose of birth defects surveillance programs is planning and prevention. Data obtained from surveillance can serve to plan promotion and prevention strategies and guide public policies (12). The case of folic acid food fortification to prevent neural tube defects demonstrates that. The surveillance data allow for the comparison and monitoring of the prevalence of neural tube defects before, during, and after the implementation of folic acid fortification of staple foods. In different places, these data have been supported the role of folic acid fortification in the decline of neural tube defects birth prevalence, therefore allowing evaluation of the effectiveness of the acid folic fortification as a preventive action (19–21).

Still from the perspective of prevention, data from birth defects surveillance programs can be used to support collective health education actions. Some regions report the prevalence of fetal alcohol syndrome using data from their birth defects surveillance programs and eventually, they look for an association between alcohol consumption during pregnancy and birth defects (12, 22, 23). These strategies allow developing education and primary prevention actions among women of childbearing age and also identifying children exposed to alcohol in the uterus that could require appropriate intervention services. Additionally, there must be successful surveillance programs to evaluate the effectiveness of prevention efforts in these situations (12, 23). It may be worth mentioning that difficulties in accurately recognizing cases of alcohol-induced birth defects have led to the emergence of specific surveillance systems for fetal alcohol syndrome, which normally screen children older than is typical in general birth defects surveillance (24–26).

### Referral to Professionals and Health Care Services

Information from birth defects surveillance programs can be used also in a familial or individual-level approach to managing the special needs of children and their families in a more suitable way. Surveillance program data may serve to refer newly identified children with birth defects for services that include specialized health care, educational and early intervention programs, and genetic counseling. Thus, affected children and their families can be connected with appropriate services promptly, contributing to establish referral networks related to medical services, community programs, and social support (27, 28). Additionally, this can facilitate access for patients with birth defects and genetic rare diseases to clinical and epidemiologic research (13, 29, 30). Furthermore, data can also be applied to evaluate the utilization of offered services (12).

Correctly predicting the request for several interdisciplinary clinics, social and educational services is crucial for children with birth defects. Based on congenital anomalies prevalence, birth defects surveillance programs can help to estimate future service demands, allowing for capacity strengthening to guarantee that necessary resources will be accessible and those appropriate professionals will be available to provide the services (31). Forecasting demand for services can be useful both in general terms and can guide the structuring of comprehensive care for people with specific congenital anomalies. To give an instance, information from birth defects surveillance programs has been provided data for planning services for children with orofacial clefts at various locations (32–34).

### Provide a Basis for Clinical Research and Human Resource Training

Finally, birth defects surveillance programs can provide data for clinical research, follow-up studies of long-term effects, and studies of economic impact, as well as provide human resource training in the surveillance and research of congenital anomalies (12, 13).

It allows carrying out population-based clinical research, which is particularly important regarding rare diseases for which big data commonly are not available (35). The development of epidemiological studies that seek to identify the patterns and causes of congenital abnormalities contributes to more adequate prevention strategies for each country. It can support research on interventions for people with birth defects and measure the outcomes and impact of these interventions on family dynamics and the quality of life of individuals. It also contributes to advancing current knowledge about diagnostics, pathophysiology, and treatment through basic research (13, 36). For instance, although thalidomide embryopathy is well-known, its pathophysiology is still not totally understood. The recent cases of children with thalidomide embryopathy identified in Brazil (37) have contributed to the development of studies that try to elucidate pathophysiologic mechanisms of this teratogen, allowing a better understanding of the susceptibility to phenotype and the development of pharmacogenomic strategies (38).

Providing education and training both in the surveillance and research of birth defects is an essential goal of the congenital anomalies' surveillance programs. For that, there is a collection of freely available courses covering surveillance methodology, coding, implementation of programs in low-resource settings, and developing strategies to prevent birth defects (39–42).

## The Benefit of Birth Defects Surveillance to Other Health-Related Programs

Birth defects surveillance data are customarily linked to vital records, like birth certificates, and thus demographic characteristics and parental survey data, such as ethnicity and education (43). It is possible to link records from birth defects surveillance with datasets from other surveillance health-related programs. These include, for example, newborn screening, early interventions, hospitalizations, and death certificates (12, 43–46).

Because of birth defects impact on child morbidity and mortality, there are many precedents of childhood mortality analyses that incorporate birth defects registry data, providing an effective mechanism for monitoring the survival and mortality risks of children with selected major birth defects, such as congenital heart disease (47), esophageal atresia (48), spina bifida (49), diaphragmatic hernia (50), and omphalocele (51).

The potential to link records and consolidate information from different databases contributes to assorted public health applications of surveillance data. To illustrate, Sales Luiz Vianna et al. investigated data from the Brazilian birth defects surveillance system, defined a more likely thalidomide embryopathy phenotype, and linked that with surveillance data from the National Leprosy Program. They showed a correlation between thalidomide prescription and that specific phenotype, reinforcing that thalidomide embryopathy should be better monitored in countries where this medication is available (37).

## Challenges for Birth Defects Surveillance Programs

Collaborative efforts must be made to standardize data collection, coding, and analysis, increasing the utility of birth defects programs globally (13, 14, 45). Promoting international cooperation is also the main question. International collaborative networks are important for improving birth defects surveillance because they contribute to the understanding of the global epidemiological setting of these disorders, besides strengthening surveillance initiatives in unassisted regions (9, 13, 52). Recent literature review about the subject identified six international congenital anomaly surveillance collaboration networks: Estudio Colaborativo Latino Americano de Malformaciones Congénitas (ECLAMC), International Clearinghouse for Birth Defects Surveillance and Research (ICBDSR), European Surveillance of Congenital Anomalies (EUROCAT), British and Irish Network of Congenital Anomaly Researchers (BINOCAR), South-East Asia Region's Newborn and Birth Defects Database (SEAR-NBBD), and Red Latinoamericana de Malformaciones Congénitas (ReLAMC) (9).

There is a need for actions to expand birth defects surveillance, prevention, and care in low and middle-income countries, sceneries often associated with poor maternal nutrition and/or exposure to infection and other teratogens, and scarce family planning programs. Particularly in conditions of limited financial resources, birth defects surveillance programs can provide an accurate estimate of the burden of congenital anomalies, which can be used to advocate for prevention and care and to also evaluate the impact of the public established actions (36, 53, 54). But even consolidated birth defect surveillance programs in the USA, Canada, and Europe suffer financial constraints that can impair their functioning. It is interesting to note that healthcare systems are sometimes reorganized to reduce costs without the procedures and data of birth defects surveillance programs being considered (55).

Additionally, some researchers advocate expanding surveillance systems to ensure that functional or developmental defects are also counted along with structural birth defects (36). The National Registry of Congenital Defects and Rare Diseases-RNDCER of Uruguay, for example, have included the mandatory notification of neonatal screening pathologies (56). Newborn screening is one of the most widely distributed population screening programs worldwide (57). Despite the discrepancy in neonatal screening programs across countries since the late 1990s, tandem mass spectrometry has been increasingly introduced into newborn screening enabling the identification of more than 30 inherited metabolic diseases, some of them with effective treatments (57, 58). However, newborn screening is useful not only for the detection of inborn errors of metabolism but also for endocrine, hematologic, immune, cardiac, and pulmonary diseases (58, 59), as well as sensory defects such as deafness (60) and visual problems (61). There is extensive discussion in the literature about the cost-effectiveness of ultrasound screening programs for birth defects (62, 63), although some congenital birth defects such as developmental dysplasia of the hip and congenital heart diseases are better identified through imaging tests (64–66). Besides that, other tools are helpful in neonatal screening such as pulse oximetry in the case of heart defects (67), the red reflex test in the case of visual problems (68), and otoacoustic emissions or auditory brainstem response in the case of hearing assessment (69). Improving the coverage of newborn screening to achieve everyone and ensuring the inclusion of diseases that can be early treated to promote secondary prevention are great challenges. That will require not only the availability of metabolic and imaging tests, but also the provision of appropriate treatment and longitudinal follow-up of children's development. This last is very important to measure the impact actions of the newborn screening program.

Recently, models of triple surveillance have been proposed to support and accelerate birth defect prevention. The concept of triple surveillance is complex and implies including and integrating the three basic domains of the causal chain, that is, from cause to disease occurrence and health outcomes. Botto and Mastroiacovo give some examples of triple surveillance for selected congenital conditions. For instance, specifically for neural tube defects, they recommend surveillance folate deficiency through blood tests, assessment of neural tube defects prevalence and lifelong disability (70).

One last point that deserves to be highlighted is that although evidence regarding Covid-19 does not suggest increased risks for congenital anomalies (71), just like many other sectors of public health and medicine, birth defects surveillance programs may be faced with organizational and methodological barriers because of the Covid-19 pandemic, requiring to reorganize and respond to a changing panorama (72).

## Conclusions

In summary, the information collected through birth defects surveillance programs is used to produce prevalence data, recognize risk factors, foster the development of research in the area, develop prevention strategies, plan for services, and referral of affected children to medical, educational, and social services. Further, there is a global tendency for congenital anomalies surveillance programs around the world to work in networks, which gives more strength to their data and conclusions. Therefore, birth defects surveillance programs constitute an important data source to guide public health actions worldwide.

## Author Contributions

DM conceived and drafted the manuscript. MS, TS, and ML helped to design the manuscript and critically revised it. All authors read and approved the final manuscript.

## Funding

This research did not receive any specific grant from funding agencies in the public, commercial, or not-for-profit sectors.

## Conflict of Interest

The authors declare that the research was conducted in the absence of any commercial or financial relationships that could be construed as a potential conflict of interest.

## Publisher's Note

All claims expressed in this article are solely those of the authors and do not necessarily represent those of their affiliated organizations, or those of the publisher, the editors and the reviewers. Any product that may be evaluated in this article, or claim that may be made by its manufacturer, is not guaranteed or endorsed by the publisher.
